# RBD-Fc-based COVID-19 vaccine candidate induces highly potent SARS-CoV-2 neutralizing antibody response

**DOI:** 10.1038/s41392-020-00402-5

**Published:** 2020-11-27

**Authors:** Zezhong Liu, Wei Xu, Shuai Xia, Chenjian Gu, Xinling Wang, Qian Wang, Jie Zhou, Yanling Wu, Xia Cai, Di Qu, Tianlei Ying, Youhua Xie, Lu Lu, Zhenghong Yuan, Shibo Jiang

**Affiliations:** Key Laboratory of Medical Molecular Virology (MOE/NHC/CAMS), School of Basic Medical Sciences and BSL-3 facility, Fudan University, Shanghai, 200032 China

**Keywords:** Vaccines, Infection

## Abstract

The pandemic of coronavirus disease 2019 (COVID-19) caused by severe acute respiratory syndrome coronavirus 2 (SARS-CoV-2) has posed serious threats to global health and economy, thus calling for the development of safe and effective vaccines. The receptor-binding domain (RBD) in the spike protein of SARS-CoV-2 is responsible for its binding to angiotensin-converting enzyme 2 (ACE2) receptor. It contains multiple dominant neutralizing epitopes and serves as an important antigen for the development of COVID-19 vaccines. Here, we showed that immunization of mice with a candidate subunit vaccine consisting of SARS-CoV-2 RBD and Fc fragment of human IgG, as an immunopotentiator, elicited high titer of RBD-specific antibodies with robust neutralizing activity against both pseudotyped and live SARS-CoV-2 infections. The mouse antisera could also effectively neutralize infection by pseudotyped SARS-CoV-2 with several natural mutations in RBD and the IgG extracted from the mouse antisera could also show neutralization against pseudotyped SARS-CoV and SARS-related coronavirus (SARSr-CoV). Vaccination of human ACE2 transgenic mice with RBD-Fc could effectively protect mice from the SARS-CoV-2 challenge. These results suggest that SARS-CoV-2 RBD-Fc has good potential to be further developed as an effective and broad-spectrum vaccine to prevent infection of the current SARS-CoV-2 and its mutants, as well as future emerging SARSr-CoVs and re-emerging SARS-CoV.

## Introduction

The outbreaks of severe acute respiratory syndrome (SARS) caused by SARS coronavirus (SARS-CoV) in 2002/2003 and those of middle east respiratory syndrome (MERS) caused by MERS coronavirus (MERS-CoV) in 2012 have highlighted the high zoonotic potential of emerging coronaviruses.^1,2^ The pandemic of coronavirus disease 2019 (COVID-19) caused by the novel coronavirus 2019 (2019-nCoV),^3^ which was also denoted as severe acute respiratory syndrome coronavirus 2 (SARS-CoV-2),^4^ or human coronavirus 2019 (HCoV-19),^5^ has resulted in more than 17 million confirmed cases and 0.66 million deaths in 216 countries, areas or territories (https://www.who.int/), endangering the global public health and economy and thus calling for the development of effective vaccines to protect at-risk populations.

Currently, more than 150 COVID-19 vaccines are under development at different stages.^6–9^ Especially, a number of COVID-19 vaccines’ phase 1/2 clinical trials have been completed, including the adenovirus-vectored vaccines (Ad5-nCoV and ChAdOx1 nCoV-19) from CanSino^10^ and Oxford University/AstraZeneca,^11^ respectively; the mRNA vaccines (mRNA-1273 and BNT162b1) from Moderna^12^ and Pfizer/BioNTech,^13^ respectively; and the inactivated vaccines (PiCoVacc and BBIBP-CorV) from Sinovac^14^ and Beijing Institute of Biological Products,^15^ respectively (https://biorender.com/covid-vaccine-tracker/). Generally speaking, all these vaccines could induce antibodies specific for spike (S) protein and receptor-binding domain (RBD), which neutralized pseudotyped and live SARS-CoV-2 infection. Some reports have shown that the neutralizing antibody titers are strongly correlated with RBD-binding IgG concentration.^16^ However, most of these vaccines developed specifically against SARS-CoV-2 infection may not be effective to prevent infection by SARS-CoV-2 with significant mutations in its spike (S) protein and the SARS-CoV and SARSr-CoVs.

Coronavirus S protein, a class I viral fusion protein consisting of S1 and S2 subunits, plays pivotal roles in virus binding, fusion and entry, and it serves as an important target for the development of vaccines and neutralizing antibodies.^17^ For example, most of the mRNA, DNA, and viral vector-based COVID-19 vaccines contain the gene encoding SARS-CoV-2 S protein.^10,12,18,19^ The RBD in S1 of SARS-CoV and SARS-CoV-2 recognizes and binds to the receptor angiotensin-converting enzyme 2 (ACE2) on the host cells and causes the conformational changes of S2 that result in virus fusion with and entry into the host cell for replication.^20,21^

Our previous studies have shown that SARS-CoV RBD contains multiple conformation-dependent epitopes and is able to induce high-titer neutralizing antibodies,^22–25^ suggesting that RBD is one of the most important targets for the development of SARS vaccine. More recently, Lu, Wei and colleagues have shown that RBD-based COVID-19 vaccine candidate is able to elicit robust RBD-specific neutralizing antibody response in the mice, rabbits and non-human primates after just a single dose injection.^26^ Vaccination with RBD has provided protection in non-human primates against SARS-CoV-2 challenge, suggesting its potential to be further developed as an effective COVID-19 vaccine for prevention of SARS-CoV-2 infection.^26^ We previously have also demonstrated that Fc fragment of human IgG in the RBD-based vaccine, RBD-Fc, can act as an important immunopotentiator to enhance the immunogenicity of RBD.^24,25,27^ SARS-CoV RBD-Fc could induce antibodies in rabbits and mice with highly potent neutralizing activity and inhibitory activity to block the binding between S1 and the host receptor ACE2.^24^ Importantly, SARS-CoV RBD-Fc could induce long-term neutralizing antibody responses that effectively protect the vaccinated mice against SARS-CoV infection.^25^

Using a similar approach, we have designed a recombinant subunit vaccine candidate, RBD-Fc, which contains RBD in S1 of SARS-CoV-2 and Fc fragment of human IgG. We found that mice immunized with the recombinant SARS-CoV-2 RBD-Fc protein produced high titer of RBD-specific antibodies, which could potently neutralize infection of SARS-CoV-2 without or with mutations and show neutralization activity against SARS-CoV and SARSr-CoVs. Therefore, this RBD-Fc-based vaccine showed high potential to be further developed as a highly effective COVID-19 vaccine.

## Results

### Recombinant RBD protein of SARS-CoV-2 exhibited good conformation and antigenicity

To obtain immunogen for immunization of mice, we constructed an expression vector encoding SARS-CoV-2 RBD and Fc fragment of human IgG (Supplementary Fig. 1a). After expression in Expi293F cells and purification with affinity chromatography, we characterized the RBD-Fc protein in both boiled and unboiled conditions using SDS-PAGE. As shown in Supplementary Fig. 1b, a single strong band with a molecular weight of ~60 kDa was noted in the boiled condition and one of ~120 kDa in the non-boiled condition, suggesting that the well-expressed RBD-Fc with high purity had formed a dimer via the Fc in the non-boiled condition. The recombinant RBD-Fc protein expressed in high yields, and the purification was high.

Using ELISA to investigate the binding activity between RBD-Fc and human ACE2 (hACE2), we found that the RBD-Fc could effectively bind to the hACE2 in a dose-dependent manner with a 50% effective dose (EC_50_) of 0.27 µM (Supplementary Fig. 1c). These results indicate that RBD-Fc exhibits proper conformation and functionality and that it can serve as an excellent immunogen.

### RBD-Fc induced strong RBD-specific IgG antibody responses in mice

**Fig. 1:**
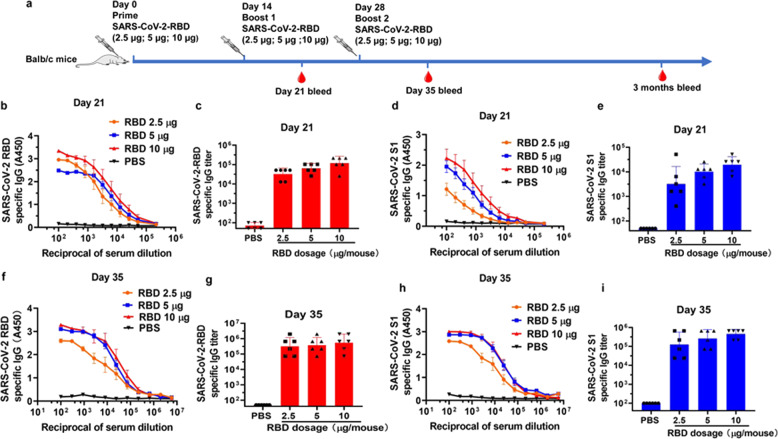
Balb/c mice immunized with RBD-Fc produced SARS-CoV-2 RBD- and S1-specific antibodies. **a** The immunization protocols. **b**, **c** Detection of the SARS-CoV-2 RBD-specific IgG in different dilutions of sera from day 21 (**b**) and the RBD-specific IgG titer in sera from day 21 (**c**) using ELISA assay. **d**, **e** Detection of the SARS-CoV-2 S1-specific IgG in different dilutions of sera from day 21 (**d**) and the S1-specific IgG titer in sera from day 21 (**e**) using ELISA assay. **f**, **g** The binding capacity of the total IgG in the sera from day 35 to SARS-CoV-2 RBD (**f**) and the SARS-CoV-2 RBD-specific total IgG titer (**g**). **h**, **i** The binding capacity of IgG in the sera from day 35 to SARS-CoV-2 S1 (**h**) and SARS-CoV-2 S1-specific IgG titer (**i**). Data shown are geometric mean ± SD from six samples. All experiments were repeated at least twice

Three different doses of RBD-Fc (10, 5, and 2.5 µg, respectively) were used to immunize mice in order to optimize the dose of antigen that could sufficiently induce antibody response. The mice were vaccinated subcutaneously three times at 14-day intervals, and the sera were collected at 7 days after each boost (Fig. 1a). The geometric mean titer (GMT) of RBD-specific IgG in sera was detected with ELISA using the protein of RBD-His, rather than RBD-Fc, to coat the wells of plates since the immunized mice could produce anti-human Fc antibodies (Supplementary Fig. 2). For the sera collected from mice on day 21 post-1st immunization with 2.5, 5, and 10 µg of RBD-Fc, the GMTs of SARS-CoV-2 RBD-specific antibody IgG are 32,254, 64,508, and 117,713, respectively (Fig. 1b, c), and those of SARS-CoV-2 S1-specific antibody IgG are 3200, 10,159 and 19,577, respectively (Fig. 1d, e), while the sera from mice treated with PBS exhibited only background level of the signal. For the sera collected on day 35 post-1st immunization with 2.5, 5, and 10 µg of RBD-Fc, the GMTs of RBD-specific antibody IgG were increased to 315,420, 378,800, and 546,323, respectively, while those of SARS-CoV-2 S1-specific antibody IgG were increased to 126,267, 262,645, and 454,914, respectively (Fig. 1f-i). These results suggest that immunization of mice 2–3 times with RBD-Fc at 2.5–10 µg could elicit similarly strong RBD-specific antibody responses.

### RBD-Fc elicited robust neutralizing antibody responses in mice against pseudotyped and live SARS-CoV-2 infection

**Fig. 2:**
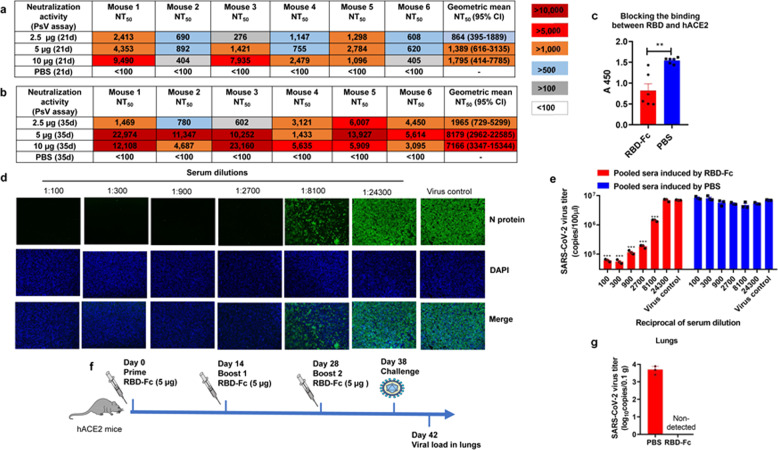
Neutralization activity of sera from day 21 and day 35 against SARS-CoV-2 PsV. **a** The NT_50_s for each mouse at day 21 using SARS-CoV-2 PsV neutralization assay. **b** The NT_50_s for each mouse at day 35 using SARS-CoV-2 PsV neutralization assay. **c** Antisera from mice immunized with 10 µg RBD-Fc inhibited hACE2 binding to SARS-CoV-2 RBD. Data shown are means ± s.e.m. from six samples. **d** Detection of SARS-CoV-2 N protein expression in cells treated with pooled sera from mice immunized with 10 µg RBD-Fc using immunofluorescence assay. **e** Detection of SARS-CoV-2 virus titer in the cell supernatant using RT-qPCR assay. Data shown are means ± s.e.m. from triplicate samples. **f** The immunization and challenge strategy of hACE2 mice. **g** The virus titers in the lungs of hACE2 mice. *P < 0.05; **P < 0.01; ***P < 0.001. All experiments were repeated at least twice

To assess whether the sera contains neutralizing antibodies against SARS-CoV-2 infection, we developed a pseudovirus (PsV) expressing S protein of SARS-CoV-2, which could simulate the SARS-CoV-2 virion to enter and infect the target cell without replication.^21,28,29^ The sera collected from mice immunized with 2.5, 5, and 10 µg of RBD-Fc at day 21 post-1st immunization potently neutralized SARS-CoV-2 PsV infection in Huh-7 cells with geometric mean 50% neutralization titers (NT_50_) of 864, 1,389, and 1,795, respectively (Fig. 2a). The geometric mean NT_50_s of the sera were increased to 1965, 8179, and 7166, respectively, at day 35 post-1st immunization (Fig. 2b). These results suggest that immunization of mice with 5 µg RBD-Fc is able to elicit highly potent neutralizing antibody responses.

We then developed a competition ELISA assay to detect whether SARS-CoV-2 RBD-specific antisera could block the interaction between SARS-CoV-2 RBD and hACE2. As shown in Fig. 2c, antibodies in the antisera from mice immunized with RBD-Fc at day 35 post-1st immunization could significantly inhibit the binding of SARS-CoV-2 RBD to hACE2, compared with the sera from mice treated with PBS, indicating that RBD-Fc-induced antibodies could neutralize SARS-CoV-2 PsV infection by blocking the binding of RBD in S protein on the surface of SARS-CoV-2 to hACE2. Aside from the ACE2 binding sites, the sera could also inhibit the binding of CR3022 antibody^30^ to SARS-CoV-2 RBD (Supplementary Fig. 3), demonstrating that the protein could induce multiple antibodies response.

Next, we tested the neutralization activity of the antisera collected from mice immunized with 10 µg RBD-Fc at day 35 post-1st immunization against live SARS-CoV-2 infection in Vero-E6 cells using an immunofluorescence assay.^31^ After the addition of the antisera at a dilution of 1:2700 or 1:8100, we found that the expression of SARS-CoV-2 N protein in the cells was almost completely, or partially, suppressed, respectively (Fig. 2d). Then, we further tested the viral RNA in the cell supernatant using an RT-qPCR assay. The results showed that the antisera could inhibit live SARS-CoV-2 infection in a dose-dependent manner with an NT_50_ of 10,523 (Fig. 2e), consistent with the result from the immunofluorescence assay.

Recently, hACE2 transgenic (hACE2-Tg) mice were used for the development of an animal model of SARS-CoV-2 infection.^32^ We, therefore, immunized hACE2-Tg mice with the same immunization strategy to investigate the protective effects of vaccination in vivo (Fig. 2f). The hACE2-Tg mice were vaccinated with 5 µg RBD-Fc three times and then challenged with SARS-CoV-2 intranasally. The lungs were collected at 4 days post-challenge to test their virus titer. As shown in Fig. 2g, the viral RNA could not be detected in the lungs from RBD-Fc-immunized mice, while relatively high viral RNA copies were detected in the lungs from PBS-treated mice.

To investigate whether the neutralizing antibody response in mice immunized with RBD-Fc could maintain at high level for a longer period of time, we tested the neutralization activity of pooled sera from mice immunized with 10 µg RBD-Fc at 3 months post-1st immunization. The results showed that pooled sera could also effectively neutralize the SARS-CoV-2 PsV infection with the NT_50_ of 2,013 (Supplementary Fig. 4). These results indicate that the RBD-Fc vaccine is able to induce relative long-term neutralizing antibody responses.

### RBD-Fc-induced antibodies inhibited SARS-CoV-2 S-mediated cell–cell fusion

**Fig. 3:**
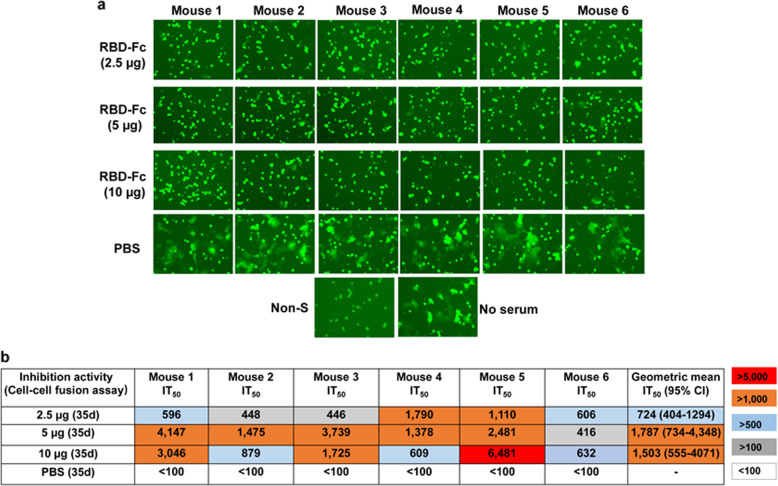
Mouse antisera inhibited SARS-CoV-2 S protein-mediated cell–cell fusion. **a** SARS-CoV-2 S protein-mediated cell–cell fusion inhibited by sera from day 35 at a dilution of 100. **b** The IT_50_s of sera from day 35 against SARS-CoV-2 S protein-mediated cell–cell fusion

Subsequently, we adapted a cell–cell fusion assay by utilizing 293T effector cells expressing SARS-CoV-2 S protein and EGFP and Huh-7 target cells expressing human ACE2^21,28^ to test whether RBD-specific antisera could block SARS-CoV-2 S protein-mediated cell–cell fusion. We found that all antisera from mice vaccinated with 2.5, 5 or 10 µg at a dilution of 1:100 could completely inhibit SARS-CoV-2 S-mediated cell–cell fusion, while the sera from the PBS-treated mice showed no significant inhibitory effect (Fig. 3a). The calculated geometric mean IT_50_ (50% inhibition titers) of the sera collected from the mice vaccinated with 2.5, 5, and 10 µg at day 35 post-1st immunization were 724 (95% CI, 404–1294), 1787 (95% CI, 734–4348) and 1,503 (95% CI, 555–4071), respectively (Fig. 3b). Consistent with the results from SARS-CoV-2 PsV inhibition assay, the 5 µg group showed inhibitory activity against cell–cell fusion similar to that of the 10 µg group, but much better than that from the 2.5 µg group, suggesting, again, that 5 µg is the optimal dose of RBD-Fc for immunization. Collectively, these results show that immunization of mice with 5 µg of RBD-Fc can elicit high titers of RBD-specific antibodies to neutralize infection of pseudotyped and live SARS-CoV-2 and inhibit SARS-CoV-2 S-protein-mediated membrane fusion.

### SARS-CoV-2 RBD-Fc could induce cross-neutralization of antibodies against infection of pseudotyped SARS-CoV-2 mutants, SARS-CoV and SARSr-CoV

**Fig. 4:**
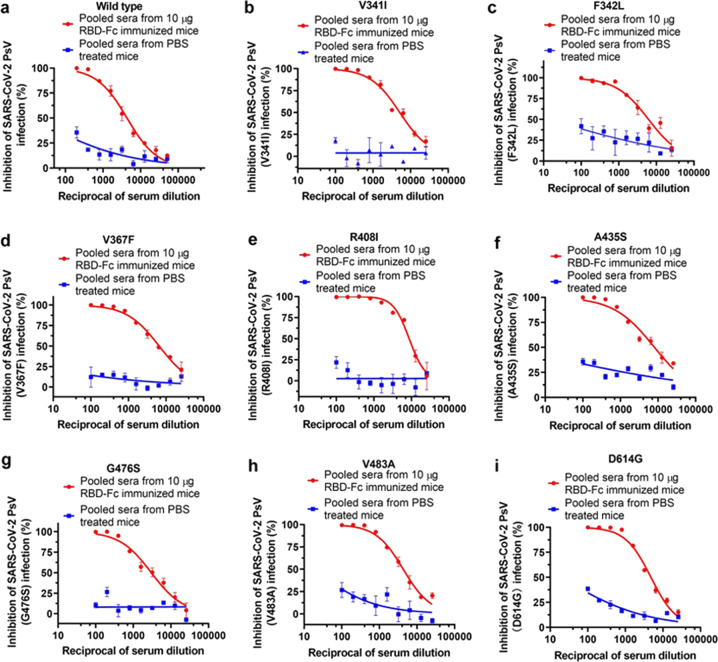
Neutralization activities of sera from day 35 against SARS-CoV-2 mutant PsVs. **a**–**i** The inhibitory effects of pooled sera from 10 µg RBD-Fc-immunized mice and pooled sera from PBS-treated mice against SARS-CoV-2 PsVs wild type (**a**), V341I (**b**), F342L (**c**), V367F (**d**), R408I (**e**), A435S (**f**), G476S (**g**), V483A (**h**) and D614G (**i**). Each point represents means ± s.e.m. from triplicate samples. All experiments were repeated at least twice

SARS-CoV-2 has been undergoing continuous mutations in its S protein, especially in the RBD region.^33,34^ which may render the virus resistant to neutralizing antibodies induced by the COVID-19 vaccines currently under development. To determine whether the mutations in RBD are resistant to the neutralizing antibodies induced by RBD-Fc, we constructed 7 SARS-CoV-2 PsVs with single natural mutation in RBD of SARS-CoV-2 S protein according to the previous report^33^ and used them to analyze their sensitivity to the neutralization activity of mouse antisera. All 7 mutants could be effectively neutralized by the pooled sera collected from mice immunized with 10 µg RBD-Fc at day 35 post-1st immunization with NT_50_ of >3000 (Fig. 4a–h). Apart from the mutations in the RBD, a lot of the natural mutants of SARS-CoV-2 have been founded. Notably, the mutant of D614G of SARS-CoV-2 has become the most dominant variant.^34^ We, therefore, also constructed D614G mutant PsV and tested the neutralization activity of the sera against D614G mutant PsV. The results showed that the pooled sera could also effectively neutralize D614G mutant PsV infection (Fig. 4i).

**Fig. 5:**
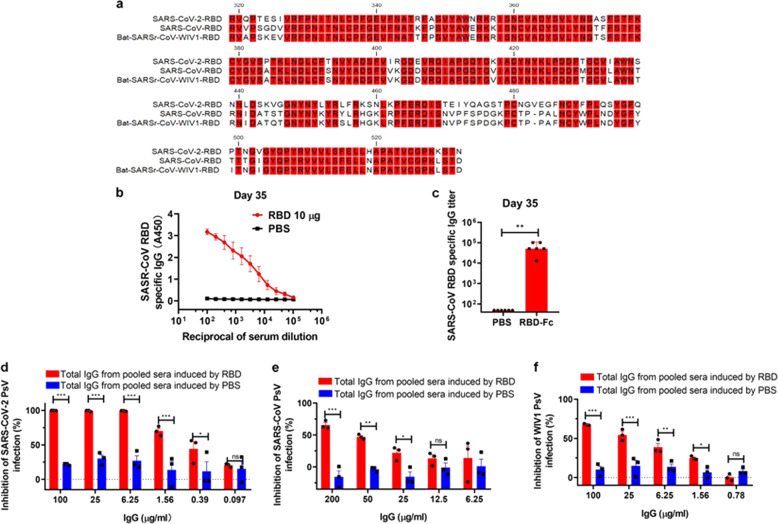
Cross-neutralization activity against SARS-CoV and SARSr-CoV. **a** The RBD amino acid sequence of SARS-CoV-2, SARS-CoV, and bat-SARSr-CoV-WIV1. **b**, **c** Detection of SARS-CoV RBD-specific IgG in different dilutions of sera (**b**) and the IgG titer to SARS-CoV RBD (**c**). **d**–**f** Neutralization effects of total IgG from the indicated sera against SARS-CoV-2 (**d**), SARS-CoV (**e**) and WIV1 (**f**). Each point represents means ± s.e.m. from triplicate samples. *P < 0.05; **P < 0.01; ***P < 0.001

Given that the RBD of SARS-CoV-2 has 74.7 and 75.2% amino acid sequence identity to the RBD of SARS-CoV and bat-SARSr-CoV-WIV1, respectively (Fig. 5a), we determined whether SARS-CoV-2 RBD-Fc-induced antibodies could cross-react with RBD of SARS-CoV using ELISA. As shown in Fig. 5b, c, the sera collected from mice immunized with 10 µg RBD-Fc at day 35 post-1st immunization could effectively bind to RBD of SARS-CoV in a dose-dependent manner with IgG GMT of 51,200. We then purified the total IgG from the pooled sera collected from mice immunized with 10 µg RBD-Fc at day 35 post-1st immunization to test its neutralization activity against SARS-CoV-2 PsV infection. We found that total SARS-CoV-2 IgG could significantly neutralize infection of SARS-CoV-2 PsV (Fig. 5d), SARS-CoV PsV (Fig. 5e), and bat-SARSr-CoV PsV (Fig. 5f), respectively, although the neutralizing activity against SARS-CoV-2 PsV turned out to be more potent than that against SARS-CoV PsV and bat-SARSr-CoV PsV. These results serve as additional evidence that SARS-CoV-2 RBD-Fc has the potential to be developed as a broad-spectrum vaccine to prevent infection by lineage B betacoronaviruses, including SARS-CoV-2 that has caused the current COVID-19 pandemic, as well as SARS-CoV and bat-SARSr-CoV that may cause the future coronavirus disease outbreaks.

### Identification of the linear epitopes in the SARS-CoV-2 RBD domain

**Fig. 6:**
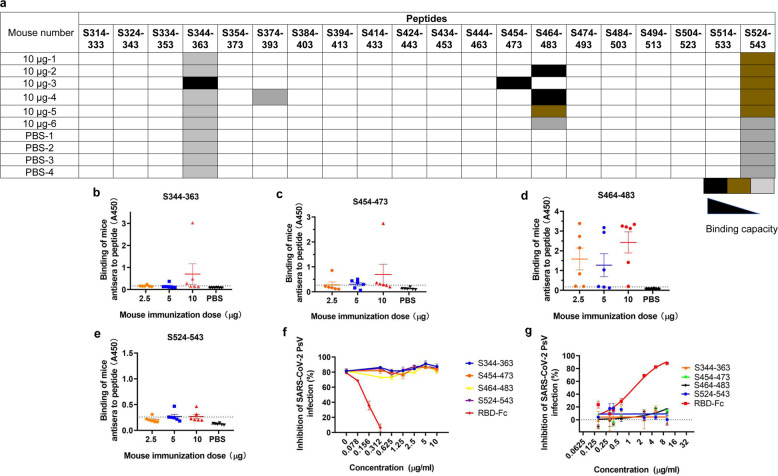
Identification of linear epitopes in the RBD region of SARS-CoV-2. **a** Detection of peptide binding to the antibodies in sera from mice immunized with 10 µg at 35 days post 1st immunization using a chip coated with overlapping peptides that covered the RBD of SARS-CoV-2. **b**–**e** ELISA assay to detect the binding capacity of mouse antisera to four positive peptides in the chip. **f** The neutralization blocking effects of the four peptides and RBD-Fc protein. Total IgG from the pooled sera immunized with RBD-Fc was mixed with the four peptides, respectively, and then the remaining neutralization effects of the IgG were detected using SARS-CoV PsV assay. **g** The inhibitory activities of the four peptides and the RBD-Fc protein against SARS-CoV-2 PsV

To investigate whether RBD-Fc-vaccinated mice could produce antibodies targeting linear epitopes in the SARS-CoV-2 RBD domain, we synthesized a group of peptides spanning the RBD region in S protein (residues 314–543). Each peptide contained 20 amino acids with 10 residues overlapping with the adjacent peptides. These peptides were precoated in a chip. The results revealed that four linear peptides in the SARS-CoV-2 RBD domain could react with the antisera (Fig. 6a). To further confirm the reactions and quantify the binding capacity, we coated the four peptides on ELISA plates to detect antibody-peptide binding in sera. Some sera induced by RBD-Fc showed higher binding capacity to the four linear peptides (Fig. 6b–e). Particularly, peptide S464-483 could strongly react with the antisera collected from mice vaccinated with 2.5, 5 or 10 µg at day 35 post-1^st^ immunization, demonstrating that S464-483 is an immunodominant linear epitope in the SARS-CoV-2 RBD domain.

We then tested whether these peptides could block the neutralizing activity of antibodies in mouse antisera. The results showed that the neutralization was not significantly affected by these peptides at the concentration as high as 10 µg/ml, while RBD-Fc at 0.078–0.312 μg/ml could effectively block the neutralization activity of RBD-specific antibody (Fig. 6f). We then tested whether these peptides could inhibit SARS-CoV-2 PsV infection and found that none of these peptides could significantly inhibit SARS-CoV-2 PsV infection, whereas the RBD-Fc protein could effectively inhibit SARS-CoV-2 PsV infection in a dose-dependent manner (Fig. 6g). These results suggest that most SARS-CoV-2 RBD-specific neutralizing antibodies recognize conformational, but not linear, epitopes. Some peptides containing linear epitopes may induce non-neutralizing antibodies, consistent with the finding of SARS-CoV RBD-specific neutralizing antibodies.^35^ When designing a COVID-19 vaccine, the epitope with the ability to induce non-neutralizing antibody or neutralizing antibody with antibody-dependent enhancement (ADE) effect should be masked with a glycan probe as previously described.^36^

## Discussion

Development of a safe and effective COVID-19 vaccine that can elicit neutralizing antibodies capable of preventing SARS-CoV-2 infection has been the global goal of controlling the COVID-19 epidemic. In consideration of the severity of the COVID-19 pandemic, several candidate vaccines have promptly entered into clinical trials. Although these candidate vaccines showed promise in induction of neutralizing antibody and T cell responses, it is still elusive whether these vaccines can indeed protect the vaccinated people from infection by SARS-CoV-2, particularly those with significant mutations in S protein, in phase III clinical trials or in future application. Compared with other vaccine types, recombinant protein-based subunit vaccines are largely considered to be safer.^37,38^ Since RBD contains the dominant neutralizing epitopes in the S protein,^39^ a vaccine consisting of RBD, rather than full-length S protein, is expected to effectively induce neutralizing antibodies and minimize the production of non-neutralizing antibodies.

It has been reported that the vast majority of neutralizing antibodies against SARS-CoV-2 target the RBD.^39^ Tai W. et al. have recently shown that the lipid nanoparticle-encapsulated (LNP) RBD-based mRNA COVID-19 vaccine elicited in mice highly potent neutralizing antibodies (NT_50_: ~10,000) against SARS-CoV-2 PsV infection.^40^ These antibodies could also cross-neutralize infection of pseudotyped SARS-CoV strains Tor2 and GD03 from humans, and SZ3 from palm civets, indicating that this RBD-based mRNA vaccine is able to induce neutralizing antibodies against both SARS-CoV-2 and SARS-CoV infection.^40^ Zhang N. et al. have also demonstrated that an LNP-encapsulated SARS-CoV-2 RBD-based mRNA vaccine (ARCoV) is able to induce in mice and non-human primates (NHPs) both neutralizing antibody response and Th1-biased cellular response, resulting in full protection against challenge of a mouse-adapted SARS-CoV-2 strain.^41^ More recently, Yang J. et al. have demonstrated that RBD-based COVID-19 vaccine could induce much higher titer of neutralizing antibody (NT_50_: ~2400) than S protein (NT_50:_ ~300) and S1 subunit (NT_50_: ~1100).^26^ Moreover, a disulfide-linked SARS-CoV-2 RBD dimer was demonstrated to enhance the neutralizing antibody titer compared to the RBD monomer.^37^ The RBD dimer might have several advantages for increasing immunogenicity, such as increasing molecular weight, better stimulation of B cells and exposure of the immunodominant neutralizing epitopes.^37^ Different from the disulfide-linked dimer approach, we conjugated the human IgG Fc fragment to the C-terminus of SARS-CoV-2 RBD to allow it to form RBD-Fc dimer, which showed excellent binding capacity to hACE2 (Supplementary Fig. 1c). In addition, the Fc fragment in RBD-based vaccine can serve as an immunopotentiator to enhance the immunogenicity of the vaccine since it promotes interaction of the vaccine with Fc receptor on the antigen-presenting cells.^27,42,43^ Although several RBD-based vaccines containing no Fc described above are effective in eliciting potent neutralizing antibody responses and protective immunity in different animal models, it is still elusive whether these neutralizing antibodies could maintain at high level for a longer period of time. Addition of Fc fragment to the RBD-based vaccine can prolong the half-life of the immunogen^44^ and promote the long-term neutralizing antibody responses. For example, our previous study has demonstrated that the titer of neutralizing antibodies in the sera of mice immunized with RBD-Fc-based SARS-CoV vaccine is able to maintain at high level (NT_50_: 580) for at least 6 months, which is much higher than that of neutralizing antibodies required for sterilizing immunity or full protection (NT_50_: 189).^44^ Most recently, we have assessed the titer of neutralizing antibodies in the sera of mice immunized with SARS-CoV-2 RBD-Fc vaccine for 3 months and found that the neutralizing antibody titer was still at high level (NT_50_: 2013, Supplementary Fig. 4), suggesting that RBD-Fc vaccine can elicit long-term neutralizing antibody responses.

In view of the severity of the COVID-19 pandemic, application of the lowest dose of vaccine and fewest boosters to produce sufficient immune response would be desirable. By injecting 2.5, 5 or 10 µg RBD-Fc to mice, we were surprised to find that 5 µg RBD-Fc is sufficient to induce high titer of RBD-specific antibodies able to neutralize SARS-CoV-2 PsV infection with geometric mean NT_50_ of 8179 and inhibit SARS-CoV-2 S-mediated cell–cell fusion with geometric mean IT_50_ of 1787, which are similar to that of mice immunized with 10 µg RBD-Fc, but significantly better than mice immunized with 2.5 µg RBD-Fc. These results had an instructive significance for the design of the optimal dose of RBD-based vaccine against SARS-CoV-2 infection. We also found that mouse antisera could block RBD binding to ACE2, demonstrating that the sera contain antibodies that can specifically bind to the key sites in RBD responsible for binding ACE2.

To date, many amino acid mutations, especially in RBD, in SARS-CoV-2 S protein have been identified and reported,^34^ raising concerns about whether the developed vaccines could also be useful against these SARS-CoV-2 mutants. Notably, most of the reported RBD-based vaccine candidates have not been tested for their neutralization activities against the emerging SARS-CoV-2 mutants. We thus successfully constructed 8 RBD mutants of SARS-CoV-2 PsV with detectable infectivity and used them to test the neutralization activity. Our results showed that the neutralization activity of the RBD-Fc-induced pooled antisera was not influenced by these natural mutations in RBD of SARS-CoV-2 PsV with NT_50_ > 3000. Especially, a previous study has demonstrated that the RBD with V367F mutation had enhanced binding affinity to ACE2.^33^ However, the RBD-Fc-induced antisera could also effectively neutralize infection of SARS-CoV-2 PsV with V367F mutation in RBD with NT_50_ of 6,532. Also, it is worth mentioning that the virus with mutation of D614G outside the RBD was more infectious,^34^ which raises the concern that the D614G mutant might be resistant to the neutralization effect of antibodies elicited by SARS-CoV-2 vaccine candidates. However, our results indicated the D614G mutant could also be effectively neutralized by the RBD-Fc-induced antisera.

Another important goal of designing COVID-19 vaccine is to make the vaccine with broad-spectrum neutralizing activity and protective efficacy against lineage B betacoronaviruses, including SARS-CoV-2, SARS-CoV and SARSr-CoV that may cause future emerging or re-emerging coronavirus diseases. Although the sequence identity of S proteins between SARS-CoV-2 and SARS-CoV is high,^45^ whether the SARS-CoV convalescent sera could cross-neutralize SARS-CoV-2 remains controversial. For example, one recent study has demonstrated that sera from the recovered SARS patents and SARS-CoV RBD-immunized mice and rabbits could strongly react with RBDs of both SARS-CoV and SARS-CoV-2 and neutralize both SARS-CoV and SARS-CoV-2 infection,^46^ while another study reported that SARS convalescent plasma could not neutralize SARS-CoV-2 infection, even though it showed strong cross-reactivity to SARS-CoV-2.^47^ Another study has shown that the monoclonal antibody S309 identified from the B cells of SARS survivor using SARS-CoV-2 S protein could bind to the RBDs of SARS-CoV-2 and SARS-CoV and, thus, cross-neutralize infection of both viruses.^48^ These studies implied that a SARS-CoV-2 RBD-based vaccine might induce cross-neutralizing antibodies against SARS-CoV and SARSr-CoV, although, the cross-neutralization activity of the antibodies elicited by most of the reported RBD-based vaccine candidates has not been shown. Here we found that the antibody in the antisera induced by SARS-CoV-2 RBD-Fc vaccine exhibited cross-neutralization activity against infection of pseudotyped SARS-CoV and a bat pseudotyped SARSr-CoV WIV1. The titer of cross-neutralizing antibodies is not high; however, it is promising for the development of RBD-based broad-spectrum vaccines against lineage B betacoronavirus infection. Consistent with the results from the pseudovirus neutralization assay, serum neutralization activity against live SARS-CoV-2 further confirmed the strong inhibitory effects with significant neutralization, even with serum diluted to 1:8,100. Most importantly, we also identified that the RBD-based vaccine could effectively protect hACE2-Tg mice against SARS-CoV-2 challenge. The protection effect of this vaccine candidate needs to be further tested in more hACE2-Tg mice, and if possible, the experiment can also be performed in rabbits and rhesus macaques.

Identification of linear immunodominant sites in RBD to discover the neutralizing or non-neutralizing epitopes is crucial for designing vaccines. We found that four peptides derived from the RBD region of SARS-CoV-2 (S344-363, S454-473, S464-483, and S524-543) could react with antibodies in the antisera from mice immunized with RBD-Fc. Interestingly, one recently published study has also reported several linear immunodominant sites in the RBD of SARS-CoV-2, including S330-349, S375-394, S450-469, S480-499, and S522-646,^49^ which partially overlap with those identified in our study. None of these peptides could significantly block the neutralization antibodies in the RBD-Fc-induced antisera (Fig. 6f), suggesting that the neutralizing epitopes in SARS-CoV-2 RBD are conformational, not linear, which is consistent with those in SARS-CoV RBD.^44^ Among these peptides, the antibody immune response against S464-483 was most obvious. Modification of this site might be a good strategy to optimize the immunogen since this site is a non-neutralizing immunodominant site. And we will furtherly use these peptides to immunize mice and test their antisera for SARS-CoV-2 neutralizing activity. If they do not neutralize or even enhance SARS-CoV-2 infection, we will optimize the RBD-based vaccine by covering these non-neutralizing immunodominant sites with glycosylation^36^ in order to design more effective and safer RBD-based vaccines.

In summary, we have designed and developed an RBD-Fc-based vaccine that can induce highly potent neutralizing antibody responses against infection of pseudotyped SARS-CoV-2, SARS-CoV, and bat SARSr-CoV-WIV1, and live SARS-CoV-2. It is effective to protect hACE2-Tg mice from SARS-CoV-2 challenge. All these results suggest that this RBD-Fc-based vaccine has a great potential to be further developed for use in humans to protect against infection of SARS-CoV-2, as well as SARS-CoV and SARSr-CoVs, which may cause future outbreaks of emerging and re-emerging coronavirus diseases.

## Materials and methods

### Cells, reagents, plasmids, viruses, and animals

HEK-293T, Huh-7, and Vero-E6 cells were obtained from the American Type Culture Collection (ATCC). The cells were cultured in Dulbecco’s Modified Eagle’s Medium (DMEM, Invitrogen, Carlsbad, CA, USA) containing 10% fetal bovine serum (FBS, Gibco, USA). Freund’s complete and incomplete adjuvants were bought from Sigma (USA). The luciferase system reagents, including cell lysis solution and substrate, were from Promega (USA). Plasmids containing pcDNA3.1-SARS-2-S, pcDNA3.1-SARS-S, pcDNA3.1-WIV1-S, pAAV-IRES-EGFP-SARS-2-S, and pNL4-3.Luc.R-E- were maintained in our laboratory. Six-week-old specific-pathogen-free (SPF) female Balb/c mice were bought from Beijing Vital River Laboratory Animal Technology Co. (Beijing, China). Eight-week-old SPF female hACE2 transgenic mice were from the Institute of Laboratory Animal Science, Peking Union Medical College, China. All the experiments related to animals were carried out according to institutional regulations (approval number 20190221–070; approval date 21 February 2019).

### Protein expression and purification

Genes encoding residues 319–532 of SARS-CoV-2 (GenBank accession number: QHD43416.1) spike protein fused with the genes of Fc or His in its N-terminal were inserted into the plasmid of PcDNA3.1. The recombinant expression plasmids were transfected into Expi293F cells, and then the cells were cultured for three days. After that, cell culture supernatants were collected and purified using affinity chromatography. The purified recombinant proteins were concentrated by ultrafiltration using Amicon Ultra-10 filters (Millipore, USA). Finally, the purified recombinant proteins were analyzed using SDS-PAGE. Briefly, 10% Tris-glycine SDS-PAGE was used to separate the proteins, and then the proteins in the gel were stained using Coomassie Brilliant Blue to visualize the protein lines. The SARS-CoV-2 S1 protein was purchased from Sino Biological (Beijing, China)

### Mouse vaccination

Balb/c mice were randomly assigned to 4 groups and each had 6 mice. Three doses of RBD-Fc (2.5, 5, and 10 µg) were diluted using PBS. Freund’s complete adjuvant was emulsified with the proteins or PBS at a volume ratio of 1:1 for the first immunization. After the prime vaccination by subcutaneous injection, the mice were boosted twice using Freund’s incomplete adjuvant, mixing RBD-Fc or PBS subcutaneously at two-week intervals. The mice were bled at 7 days post each boost, and the sera were separated and inactivated at 56 °C for 30 min.

### ELISA

ELISA was used to detect the binding between RBD-Fc and human ACE2. RBD-Fc protein (2 µg/ml) was coated on the ELISA plates at 4 °C overnight. Then the plates were blocked using blocking buffer (PBST containing 5% BSA) at 37 °C for 2 h. A series of concentrations of human ACE2 protein was added to the plate and incubated at 37 °C for 2 h. After 4 times of washing using PBST, rabbit anti-human ACE2-specific antibody (Sino Biological, Beijing, China) was added to the plate and incubated for 2 h at 37 °C. Subsequently, HRP-conjugated goat anti-rabbit IgG (Dako, Denmark) was added to the plates and incubated for 1 h at 37 °C. The reaction was finally visualized by adding 3,3’,5,5’-Tetramethylbenzidine (TMB) and then stopped by H_2_SO_4_. A microplate reader (Infinite M200PRO, Switzerland) was used to collect the data at the absorbance of 450 nm.

The antibody response in the mice immunized with RBD-Fc was performed by coating RBD-His protein (1 µg/ml) on ELISA plates, followed by adding serially diluted SARS-CoV-2 RBD-Fc immunized mouse sera and HRP-conjugated goat anti-mouse IgG (Dako). Titers were expressed as the reciprocal of the last dilution exhibiting A450 ≥ 2.1-fold of the background values.

A blocking ELISA was used to detect whether the immunized mouse sera could block the binding between SARS-CoV-2 RBD and hACE2 and the binding between SARS-CoV-2 RBD and CR3022. ELISA plates precoated with RBD-His were incubated with the sera at 37 °C for 1 h first before hACE2/CR3022 was added. Then, the protocol was done as described above.

Neutralization of pseudotyped SARS-CoV-2, SARS-CoV and WIV1 infection Production of the SARS-CoV-2 PsV, SARS-CoV PsV and WIV1 PsV was conducted as previously described.^21,45^ Briefly, the plasmids of PNL4–3.luc.RE and pcDNA3.1-SARS-CoV-2-S/pcDNA3.1-SARS-CoV/pcDNA3.1-WIV1 were cotransfected into HEK293T cells using a transfection reagent of VigoFect (Vigorous Biotechnology, China). The supernatant containing pseudotyped particles was collected at 60 h post-transfection. The pseudotyped viruses were preserved at −80 °C until use.

The plasmid of pcDNA3.1-SARS-CoV-2-S was used as a template to replace the relative amino acids using a site mutation kit (Yeasen, China). Then, the SARS-CoV-2 mutant PsVs were produced as described above.

Sera neutralization activities in the pseudovirus system were assessed as previously described.^21,45^ Briefly, 1 × 10^4^ /well of Huh-7 cells were seeded into a 96-well plate. After 8 h of culture, one of the PsVs (SARS-CoV-2 PsV, SARS-CoV PsV or WIV1 PsV) was mixed with a serial dilution of sera and incubated at 37 °C for 0.5 h. Then, the mixtures were transferred into Huh-7 cells, and the supernatant was replaced with fresh DMEM containing 2% FBS after 12 h. The cells were continuously cultured for 48 h, and then the luciferase activity was detected using a Firefly Luciferase Assay Kit (Promega, Madison, WI, USA).

### Cell–cell fusion assay

Neutralization activities of the sera were evaluated using S-mediated cell–cell fusion assay as previously described with a simple modification.^21,28^ Briefly, HEK293T cells were transfected with the plasmid of pAAV-IRES-EGFP-SARS-2-S or pAAV-IRES-EGFP. When the EGFP was effectively expressed in the effector cells (293T/S/GFP), the mixtures of the effector cells (1 × 10^4^ cells/well) and the diluted sera from mice treated with RBD-Fc or PBS were added into the target Huh-7 cells (2 × 10^4^ cells/well). After incubation for 4 h, the fused and unfused cells in five randomly selected fields were counted using an inverted fluorescence microscope (Nikon Eclipse Ti-S).

### Extraction and purification of mouse total IgG

Total IgG from 10 µg RBD-Fc- treated mice pooled sera or PBS-treated mice pooled sera was purified using protein G affinity chromatography (GE Healthcare, USA). The eluted IgG was concentrated by ultrafiltration using Amicon Ultra-30 filters (Millipore). The neutralization assay was conducted as described above.

### Live SARS-CoV-2 neutralization assay in BSL3

The live SARS-CoV-2 neutralization assay was done as previously described with simple modification.^31^ Vero-E6 cells were seeded in a 96-well plate (10000 cells/well) at 24 h before infection. The sera were serially diluted and mixed with 0.1 MOI SARS-CoV-2 at 37 °C for 0.5 h, and then the mixtures were added into the cells and further cultured for 48 h. The supernatants were collected to conduct the RT-qPCR assay. The cells were fixed by 4% paraformaldehyde to conduct the immunofluorescence assay. For the RT-qPCR assay, total RNA in the supernatants was extracted using Trizol LS (Thermo) first. Then, RT-qPCR was performed using a One-Step PrimeScrip RT-PCR Kit (Takara, Japan) with primers and probe as follows: SARS-CoV-2-ORF1ab-F: CCCTGTGGGTTTTACACTTAA, SARS-CoV-2-ORF1ab-R: ACGATTGTGCATCAGCTGA, SARS-CoV-2-ORF1ab-probe: 5′-FAM-CCGTCTGCGGTATGTGGAAAGGTTATGG-BHQ1-3′. For the immunofluorescence assay, the cells were treated with 0.1% Triton X for 15 min at room temperature and then blocked by 3% BSA. After that, the anti-N antibody (1:1000) was added to the cells and incubated at 4 °C overnight. Finally, Alexa Fluor donkey anti-mouse IgG 488-labeled antibody (Thermo) was added to the cells and incubated at 37 °C for 1 h. After staining with DAPI, the cells were imaged with fluorescence microscopy.

### hACE2 transgenic mouse challenge experiment

A total of six hACE2 mice were randomly assigned to two groups, and each group contained three mice. The hACE2 mice immunization strategy was done as described above. After the third immunization, the mice were challenged with 10^5^ TCID_50_ SARS-CoV-2 intranasally. After 4 days of infection, the mice were sacrificed, and the lungs were collected and homogenized in Trizol (Takara). Next, the total RNA in the lungs was extracted. RT-qPCR was used to determine SARS-CoV-2 virus titer in the lungs.

### Pepscan assay

A total of 20 peptides were synthesized by GL biochem LTD (Shanghai, China). The peptide sequences were as follows: S314–333: QTSNFRVQPTESIVRFPNIT; S324–343: ESIVRFPNITNLCPFGEVFN; S334–353: NLCPFGEVFNATRFASVYAW; S344–S363: ATRFASVYAWNRKRISNCVA; S354–S373: NRKRISNCVADYSVLYNSAS; S374–393: FSTFKCYGVSPTKLNDLCFT; S384–S403: PTKLNDLCFTNVYADSFVIR; S394–413: NVYADSFVIRGDEVRQIAPG; S414–S433: QTGKIADYNYKLPDDFTGCV; S424–S443: KLPDDFTGCVIAWNSNNLDS; S434–S453: IAWNSNNLDSKVGGNYNYLY; S444–S463: KVGGNYNYLYRLFRKSNLKP; S454–S473: RLFRKSNLKPFERDISTEIY; S464–S483: FERDISTEIYQAGSTPCNGV; S474–S493: QAGSTPCNGVEGFNCYFPLQ; S484–S503: EGFNCYFPLQSYGFQPTNGV; S494–S513: SYGFQPTNGVGYQPYRVVVL; S5044–S523: GYQPYRVVVLSFELLHAPAT; S514–S533: SFELLHAPATVCGPKKSTNL; and S524–S543: VCGPKKSTNLVKNKCVNFNF. The peptide blocking neutralization assay was done as described above with simple modification. Briefly, 4 µg/ml of total IgG extracted from the pooled sera induced by 10 µg RBD-Fc were incubated with increasing concentration of peptides at 37 °C for 30 min. After that, the mixture of peptides and antibody was incubated with the SARS-CoV-2 PsV. Then, the mixture of peptides, antibody, and SARS-CoV-2 PsV was added to the Huh-7 cells. Luciferase activity was detected using a Firefly Luciferase Assay Kit (Promega).

### Statistics

All statistical analyses were carried out with GraphPad Prism software. *P* < 0.05 was considered significant. *P < 0.05; **P < 0.01; ***P < 0.001. Student’s unpaired two-tailed *t*-test was used to compare the binding capacity to ACE2 between RBD-Fc-induced sera and PBS-induced sera. One-way ANOVA was used to compare the neutralization activity between IgG induced by RBD-Fc and IgG induced by PBS and live SARS-CoV-2 neutralization between RBD-Fc-induced sera and PBS-induced sera.

## Supplementary information

Liu Z RBD-Fc based vaccine supplementary material

## Data Availability

The data sets of the study are available from the corresponding authors upon reasonable request.
