# Supplementary material for: RBD-Fc-based COVID-19 vaccine candidate induces highly potent SARS-CoV-2 neutralizing antibody response
Source: Signal Transduct Target Ther. 2020 Nov 27;5:282. doi: 10.1038/s41392-020-00402-5 (PMC7691975; doi:10.1038/s41392-020-00402-5)
Supplement: Supplementary file 1 — Liu Z RBD-Fc based vaccine supplementary material [file 41392_2020_402_MOESM1_ESM.docx]

Supplementary Materials for

RBD-Fc-based COVID-19 vaccine candidate induces highly potent SARS-CoV-2 neutralizing antibody responses

Zezhong Liu^1, 2,^ Wei Xu^1, 2^, Shuai Xia^1, 2,^ Chenjian Gu^1, 2,^ Xinling Wang^1,^ Qian Wang^1,^ Jie Zhou^1^, Yanling Wu^1^, Xia Cai^1^, Di Qu^1^, Tianlei Ying^1^, Youhua Xie^1^, Lu Lu^1^*, Zhenghong Yuan^1^*, Shibo Jiang^1^*.

Correspondence to: [shibojiang@fudan.edu.cn；lul@fudan.edu.cn；zhyuan@shmu.edu.cn](mailto:shibojiang@fudan.edu.cn；lul@fudan.edu.cn；zhyuan@shmu.edu.cn)

**This PDF file includes:**

Figures. S1 to S4

Figure S1


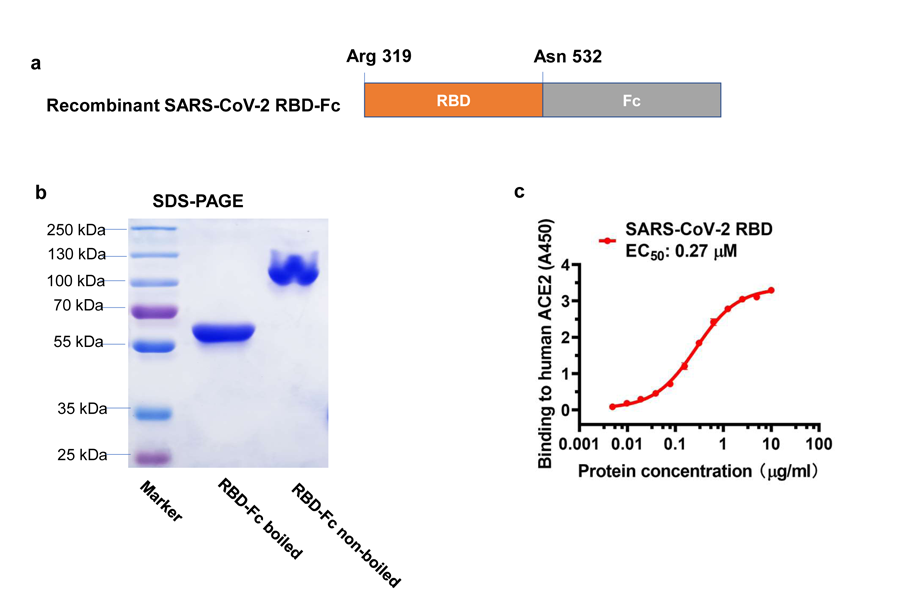


**Fig. S1. Preparation and identification of the immunogen.** **a** Construction of the RBD protein fused with human IgG Fc fragment. **b** SDS-PAGE to visualize the RBD proteins in boiled or non-boiled condition. **c** Evaluation of the binding capacity between RBD-Fc and hACE2. Data are means ± s.e.m. from triplicate samples.

Figure S2


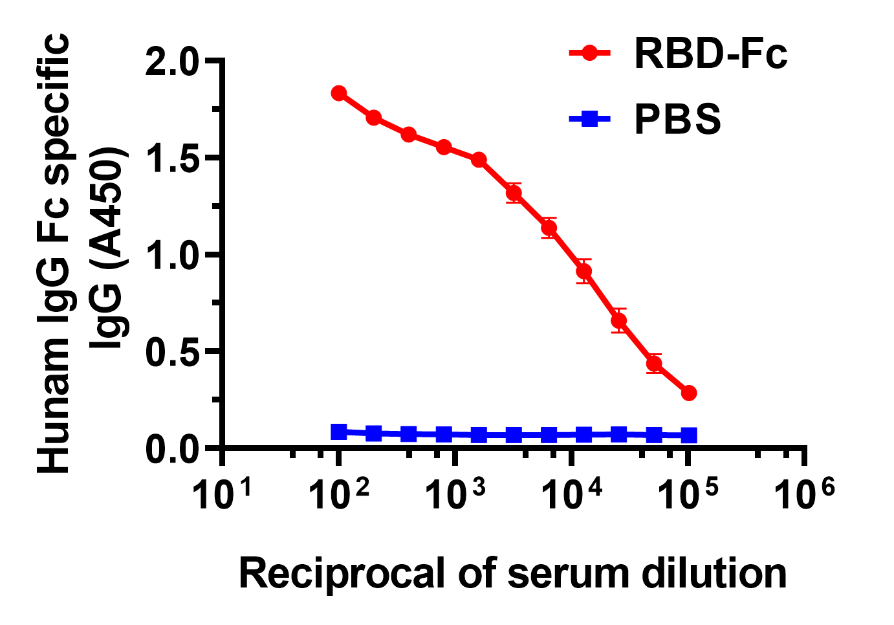


**Fig. S2. Detection of the human IgG Fc-specific IgG in sera at different dilutions.** Sera from RBD-Fc immunized mice and PBS-treated mice were used to evaluate the binding capacity to the human IgG Fc. Data shown are means ± s.e.m. from six samples.

Figure S3


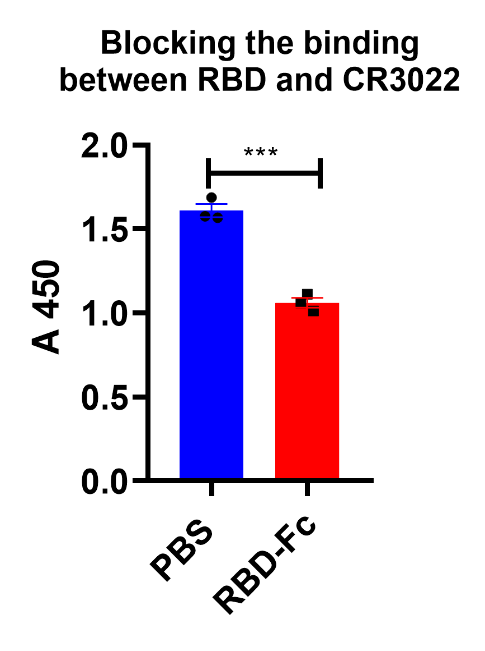


**Fig. S3. Pooled sera from mice immunized with 10 µg RBD-Fc inhibited CR3022 binding to SARS-CoV-2 RBD.** Sera were added to the ELISA plate coated with RBD-His before the addition of CR3022 antibody. After incubating with the HRP-conjugated goat anti-human IgG, TMB was used to show the reactions. Data shown are means ± s.e.m. from triplicate samples.

Figure S4


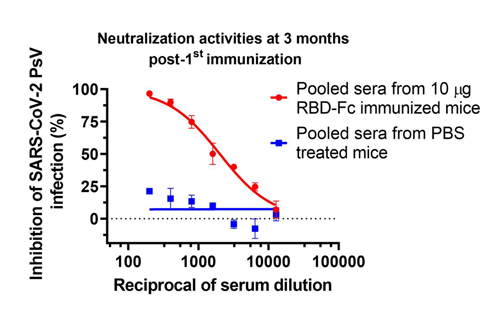


**Fig. S4. The neutralization activity of pooled sera from mice immunized with 10 µg RBD-Fc at 3 months post-1st immunization.** The neutralization activity of the sera was tested using the SARS-CoV-2 PsV assay. Each point represents means ± s.e.m. from triplicate samples.
